# Depressive Symptoms and Urbanization - A Cross-Sectional Network Analysis

**DOI:** 10.1192/j.eurpsy.2024.218

**Published:** 2024-08-27

**Authors:** D. Ochnik, B. Buława, P. Nagel, M. Budziński, M. Gachowski

**Affiliations:** ^1^Clinical Psychology; ^2^Architecture; ^3^Project Management, Academy of Silesia, Katowice, Poland

## Abstract

**Introduction:**

With increasing urbanization, more people are exposed to mental health risk factors stemming from the urban social or physical environment. However, research on urbanization and depression is not clear.

**Objectives:**

This study aimed to explore environmental and social factors with depression symptoms in view of a network theory of mental health disorders.

**Methods:**

The study was conducted among a representative sample of 3,296 habitants of Metropolis GZM (63% of women)– the most urbanized region in Poland. The measurements used were PHQ-9, UCLA, Neighbourhood Cohesion (Neighbourhood Belonging and Social Cohesion), REAT 2.0 (Quality of architecture in neighborhood area), distance and frequency use of green public areas, Self-Rated Health, Physical Activity, size of place of residence per person.

**Results:**

The prevalence of depression risk in villages (*N*=713), towns under 20,000 (*N*=219), towns (under 99,000; *N*=823), and cities (under 300,000; *N*=1541) was 44.2%, 44.7%, 39.2%, and 34.9% respectively.

The depression nodes with the highest centrality degree and expected influence were PHQ9 (suicidal thoughts), PHQ2 (feeling depressed), and neighborhood belonging. Living in a more urbanized area (UA) had a smaller centrality degree in the network. Edges between PHQ9 and environmental factors were mediated by loneliness (UCLA). Poor architectural conditions (REAT) were linked positively with neighborhood belonging and adversely with social cohesion. Living in UA was negatively related to PHQ9, PHQ5 (eating control), and PHQ2, social cohesion, and green area distance, while positively to PHQ7 (problems with being focused), poor physical health, REAT, and neighborhood belonging (Figure 1).

**Conclusions:**

Living in a city is negatively related to the most central depression symptoms. Even though social cohesion is negatively linked to UA, neighborhood belonging is higher in more urbanized areas.

The balance between detrimental environmental factors and those that protect mental health requires a better understanding of the interaction between urban living and depression.

**Disclosure of Interest:**

None Declared

